# Evaluation of Mode-I Fracture Toughness of Asphalt Mixtures with Symmetric Geometry Specimen at Intermediate Temperature

**DOI:** 10.3390/ma15144977

**Published:** 2022-07-17

**Authors:** Juan Francisco Pérez-Landeros, Pedro Limón-Covarrubias, José Roberto Galaviz-González, David Avalos-Cueva, María de la Luz Pérez-Rea, Miguel Zamora-Palacios

**Affiliations:** 1 Faculty of Engineering, Autonomous University of Queretaro, Santiago de Querétaro 76010, Mexico; franciscopl909@hotmail.com (J.F.P.-L.); perea@uaq.mx (M.d.l.L.P.-R.); 2 Department of Civil Engineering and Topography, University of Guadalajara, 1421 Blvd. Marcelino García Barragán, Guadalajara 44430, Mexico; ingenieria_limon@hotmail.com (P.L.-C.); david.avalos@academicos.udg.mx (D.A.-C.); miguel.zamora@cucei.udg.mx (M.Z.-P.)

**Keywords:** asphalt cracking, direct tension test, cracking model, finite element model

## Abstract

Mode I fracture (tensile type) is the common cracking mode of asphalt pavements, which is caused by thermal cyclic loading or traffic. Some studies allow the analysis of the fracture modes by means of standardized tests, some of which are limited, difficult, with little repeatability or do not generate an adequate tension state. In this paper, mode I fracture toughness of asphalt mixtures with symmetric geometry specimens at intermediate temperature is evaluated. Experimental results from direct tension test and simulations on asphalt mix specimens subjected to intermediate temperatures of 10, 20 and 30 °C, mode I load rates (0.5, 1 and 2 mm/min) and notches (2 and 3 cm) were compared to find the variables that reflect the operating conditions of the asphalt mix. Results showed that shear stresses are 8.12% lower in the simulations with respect to the tests, while the load-deformation curves show 30% and 35% variation, where the temperature of 20 °C, the notch of 2 cm and the loading speed of 1 mm/min are the conditions that best represent the stress state of the test; moreover, it manages to consider the elastic and viscous components of the material.

## 1. Introduction

In recent decades, the demand for asphalt pavement has increased due to the increase in the number of vehicles and transportation, exceeding the supply of existing infrastructures [1]. Thus, the growth and development of a country requires a road communication network [2], as it drives the country’s economy and the transportation of goods and people [3,4]. Asphalt pavement is often used in the construction of roads and parking lots [5] that allow continuous circulation in space and time while providing adequate levels of safety and comfort [6]. However, according to Rashadul Islam et al. [7] and Rasol et al. [8], existing pavements are no longer safe and comfortable, as they deteriorate over time, resulting in potholes, ruts, cracks, and other depressions. Similarly, Bevacqua et al. [9] found that deterioration depends on water infiltration, weather conditions, repetitive traffic loading, stiffness gradients, construction problems, and the intrinsic and fracture properties of asphalt mixtures and pavement structures, which all clearly show the structural degradation of the pavement [4,7,8,10,11,12,13,14,15,16,17,18]. This occurs because asphalt is a viscoelastic material and its behavior depends on temperature, i.e., the binder becomes viscous as temperature increases; otherwise, the asphalt turns brittle and susceptible to cracking [19]. Therefore, cracking is one of the main deterioration mechanisms of asphalt pavements [20], thus when an asphalt layer has a crack on its surface, it is usually subjected to opening mode (Mode I) loading [21], in which fatigue cracking is induced by normal stresses [4,12,14,15,18,22]. Fatigue cracking can be identified by two mechanisms. The first is bottom-up cracking (BUC), which propagates from the base layer to the surface and is caused by stresses and strains in the base layer. For the second mechanism, cracks initiate and propagate from the surface towards the base (top-down cracking, TDC) [4,9,12,14,15,18,23,24]; in this case, the mechanical properties of the mix change due to the daily or seasonal temperature gradient or vehicle passage over the asphalt layer [25].

According to Chen et al. [4], cracks occur in the asphalt binder and/or at the asphalt binder/aggregate interface. Lytton et al. [26] report that an asphalt mixture can resist fatigue damage caused by loading and thermal conditions by using a higher asphalt content, while variations in the gradation of an asphalt mix are regularly subjected to changes in the stress states, which complicates construction processes [27] since an optimal volumetric concentration can increase the fracture energy required to induce pavement failure [28]. Coarse aggregate grading reduces the onset of cracking due to interference with the rearrangement of the mix under compressive loads [29], while aggregate size sensitivity can provide a high bonding capacity in the mix, with dense-grained materials being the most recommended [30] since they provide an opposition to the fracture energy. Water filtration and the addition of air, an oxidation process, is generated in the mixture as the number of voids increases [31,32], accelerating the aging processes of the pavement and increasing its fragility, decreasing its fracture resistance [33,34], and increasing the probability of cracking failure due to thermal changes [35,36]. Therefore, environmental agents can change the mechanical characteristics of the pavement [37]; as the temperature decreases, the mixture becomes stiffer, reducing its elasticity [38,39] and making it prone to cracking more than less stiff ones [40,41,42,43]. This is because temperature impacts the viscoelastic and stress state behavior, resulting in fracture initiation or propagation [44]. In addition, the thickness of asphalt layers influences cracking [45]. TDC is the cause of cracking in thick layers, whereas the BUC predominates in thin layers [12,15].

Dave and Behnia [46] found that the fracture mechanism in a heterogeneous viscoelastic composite with thermorheological properties, such as an asphalt mixture, is complex and cannot be simulated properly with constitutive laws of linear fracture mechanics. According to Canestrari and Ingrassia [12], the most significant cracking models are based on fracture mechanics (which better describes the propagation phase) or continuous damage mechanics (which are more appropriate for studying the initiation phase). In recent years, significant advances have been made in the analysis of fracture behavior in pavement [47,48]. Numerous investigations have been carried out to evaluate cracking in pavement using standardized experimental tests, indirect and nondestructive methods [8,49], cohesive zone (CZ) simulations [16], discrete element models (DEMs) [5], extended finite element models (XFEMs) [7], and finite element models (FEMs) [8,14].

The standardized tests for analyzing fractures in pavement allow for the analysis of the different fracture modes of asphalt pavement; however, most of these tests are limited [50]. Hu et al. [51] mention that there is not enough evidence to be considered reliable because they involve a difficult procedure, with little repeatability and do not produce an appropriate stress state. However, Zhou et al. [41] used the bending beam fatigue test (BBF), the indirect creep and tensile strength test (IDT-CST), the overlay test (OT), the disk compact tension test (DCT), and three versions of the semicircular bending test (SCB) to determine cracking behavior to validate an indirect asphalt cracking test (IDEAL-CT). This test combines seven desirable features (simplicity, practicality, efficiency, test equipment, repeatability, sensitivity, and correlation with field performance) of NCHRP 9-57. These tests have been used to estimate cracking in asphalt mixtures modified with SBS and MA polymer [52], as well as other mixtures containing different percentages of reclaimed asphalt pavement (RAP) material in combination with glass fibers, at different temperatures (−15, 0 and 15 °C) and with different notch lengths (25.4, 31.7 and 38.1 mm) [53]. Additionally, Wang et al. [54] evaluated cracking in asphalt mixtures with SBS polymer and RAP.

Indirect and nondestructive methods have been developed due to cracks in road pavements being a common source of damage that can be difficult to detect with visual inspections [8]. One of these is digital image processing (DIP), allowing the detection and monitoring of cracks and deformations by comparing changes in pixels of a deformed image from an undeformed one [49]. Another method is ground penetrating radar (GPR), which allows for evaluating the orientation of cracks with respect to the antenna dipoles. In addition, the falling weight deflectometer can be used to evaluate pavement stiffness and estimate the degree of cracking, but it is limited as it only applies for BUC [8].

Finite element models (FEMs) are important for fracture mechanics analysis since they clearly show the stress states present in asphalt mixtures leading to failure [55]. In these environments, the material parameters are predefined under loading, temperature and mechanical conditions, showing the representation of the situations to which they may be exposed by minimizing the manufacturing of physical samples [56], proving to reach stress states with significant realism once they are conceived in three-dimensional environments. Furthermore, FEM models have been applied to analyze pavements [4], validated with data from experimental fracture tests [18,46], and the predicted responses are affected by geometric dimensions, material properties, and load types [4]. Chen et al. [4] introduced pavement structure and boundary conditions into the simulation. Elseifi et al. [14] used the cohesive zone (CZ) model to simulate failure mechanisms through damage propagation, while Kim and Buttlar [16] used the CV model to measure low-temperature cracking behavior. Liu et al. [5] combining the DEM and FEM to study microscopic crack propagation with scanning electron microscopy (SEM). Rashadul et al. [7] used the XFEM model to examine crack propagation in pavement systems with and without pre-existing cracks.

Literature shows that the Mode I fracture toughness of asphalt mixtures is important to analyze because it generates problems for pavements. Different tests have been designed to measure fracture toughness directly, but some procedures are limited, difficult or have low repeatability. Indirect methods do not allow for preventing cracking. Numerical models have been proven to be able to simulate real conditions to predict cracking but are becoming increasingly complex and require more computational resources. A simple experimental test which does not require costly equipment, provides adequate repeatability, and has a numerical model to stimulate the test can reduce analysis time and cost. 

Therefore, the objective of this paper is the evaluation of mode I fracture toughness of asphalt mixtures with symmetric geometry specimens at intermediate temperature. For this purpose, circular specimens were cut and notched at their edges to induce a fracture in the central notch, generating a symmetric geometry. Experimental results from direct tension tests and simulations on asphalt mix specimens subjected to intermediate temperatures of 10, 20 and 30 °C, mode I load rates (0.5, 1 and 2 mm/min) and notches (2 and 3 cm) were compared to find the variables that reflect the operating conditions of the asphalt mix. Simulations were carried out in the ANSYS environment, in which the three-dimensional solid finite element SOLID85 was used. Based on the results, simulations were performed and compared with the experimental test results.

## 2. Materials and Methods

To achieve the objective of this research project, it was necessary to perform the work in two stages. In the first stage, the cracking of a hot mix asphalt (HMA) was evaluated by a direct tension test. During this stage, different temperatures, load application rates, and notch lengths were considered to determine the ideal conditions for these tests.

For the second stage, to simulate the cracking behavior of the asphalt mixtures, a finite element model (FEM) was used in the ANSYS environment, which considers Hooke’s law and Maxwell’s viscoelastic model, including the SOLID185 finite element with three degrees of freedom at each node that supports plasticity, hyperplasticity, tensile stiffness, creep, large deflection, and strain capacities. Thus, the purpose of this stage was to integrate the ideal conditions derived from the direct tension experimental test, thus providing a tool to predict fatigue cracking in asphalt mixtures subjected to mode I fracture at intermediate temperatures.

### 2.1. Aggregate Characteristics

The aggregate used was of basaltic origin and was 100% crushing product. Two material gradations were used: gravel with a maximum size of 3/4″ and sand with a maximum size of 2.36 mm. The aggregate material was sampled in accordance with the Secretariat of Communications and Transportation [57] regulation M-MMP-4-04/02, the characteristics of which are shown in Table 1 and Table 2.

### 2.2. Preparation of Specimens for the Direct Tension Test

**Figure 1:**
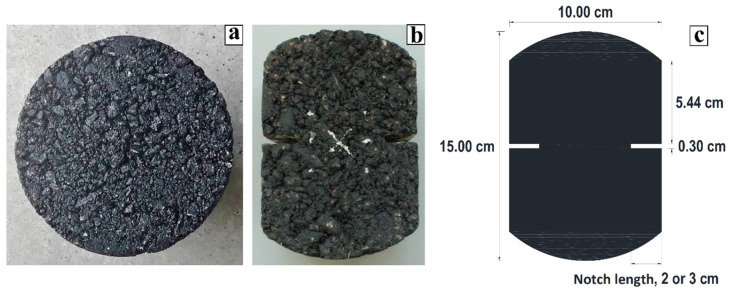
(**a**) Specimen produced with HMA asphalt mix, (**b**) cuts and notches in the specimen, (**c**) length of the notches.

As shown by Zhou et al. [41] and considering that described by Aliha et al. [19], the geometry of the test specimens is generally circular, since asphalt samples are usually manufactured by a cylindrical Superpave Gyratory Compactor (SGC), or are extracted from the field using cylindrical coring devices. Therefore, in this work, a hot mix asphalt (HMA) was designed using a cylindrical laboratory test specimen that was 15 cm in diameter and 5 cm thick (see Figure 1a) based on the methodology of the Superpave method, as mentioned in Kennedy et al. [58].

Additionally, Aliha et al. [19] mentioned that the symmetry of the geometry results in crack initiation and propagation occurring under mode I (crack opening without slip). Hence, to obtain a symmetrical geometry, samples were cut 2.50 cm vertically from the edges towards the center, while notches were made on the horizontal midline to induce a fracture in the center notch (see Figure 1b,c). In addition, Eghbali et al. [20] concluded that a notch/thickness ratio of 0.4 (2 cm notch/5 cm thick) is adequate, as the results are similar to the SCB test. Therefore, to verify the above, the lengths of the notches considered in the experimental design were 1, 2, and 3 cm (see Figure 1c); however, due to the complexity of making the 1 cm notches with the cutting equipment, these lengths were discarded. Finally, the test specimens of the current investigation are prepared using asphalt mix compacted at the laboratory, whose geometry is symmetrical, as shown in Figure 1. 

On the other hand, Fattahi Amirdehi et al. [59] measured the fracture toughness in mode I with a constant rate of 3 mm/min at −15 °C, while Fuan et al. [21] did so from 1 to 6 mm/min at −15 °C a −25 °C. From the above, they concluded that lower temperatures and higher loading rates result in elastic behavior; in contrast, higher temperatures or lower loading rates result in viscous behavior of the binder. Therefore, to find the viscoelastic behavior of the asphalt mixture, the specimens were subjected to the direct tension test at different load application rates (0.5, 1, and 2 mm/min) and test medium temperatures (10, 20, and 30 °C). This temperature range was chosen because the fatigue cracking phenomenon occurs in this range—in which neither excessive stiffness (thermal cracking) nor high deformation (rutting) dominates. This experimental design is shown in Table 3.

### 2.3. Direct Tension Test

**Figure 2:**
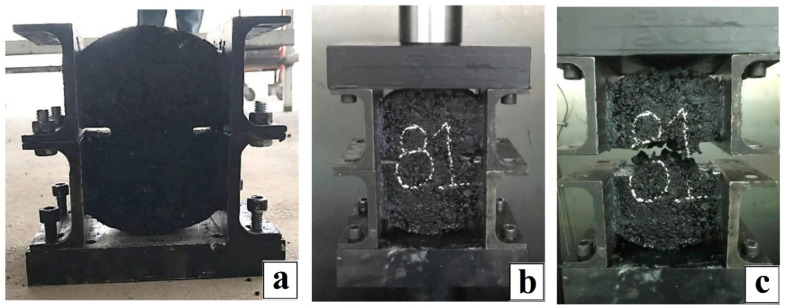
(**a**) Specimen clamping system, (**b**) direct tension test clamping system on the GCTS machine, (**c**) specimen fracture produced in the direct tension test.

The purpose of the direct tension test was to evaluate the mode I fracture toughness of asphalt mixtures at medium temperatures. For this test, it was necessary to design and adapt a system to clamp the asphalt specimen in a GCTS universal machine (see Figure 2a), which was used to perform the tests at the controlled deformation rates and test temperatures shown in the experimental design in Table 3. Thus, due to the multiple experimental variables present in the direct tension test and to establish the relevance, repeatability, and sensitivity of the test, the experimental design was performed three times. As a result, it was possible to evaluate the mode-I fracture toughness of asphalt mixtures with symmetric geometry specimen at intermediate temperature.

Figure 2b shows the clamping system placed on the GCTS universal machine, where the two flat faces of the specimen were clamped by plates and a Devcon^®^ epoxy resin has a tensile strength of 143 kg/cm^2^ and showed excellent adhesion and holding of the specimen without sample slippage. Thus, due to the epoxy’s high tensile strength and adhesion, it does not significantly affect the fracture toughness of the specimen. The bottom section of the clamping system is connected to the immovable pedestal of the GCTS machine by clamping screws. This bottom section restricts the horizontal and vertical displacement of the specimen. On the other hand, the top section confines the specimen horizontally and is connected to the GCTS head. Finally, the top clamp moved upward as the machine applied the upward load, limiting the lateral displacement. With this movement, a load was applied, inducing in-plane fractures in the notch and causing specimen failure (see Figure 2c).

### 2.4. Finite Element Model to Simulate the Direct Tension Test

Simulations are an experimentation technique that recreate certain aspects of reality, enabling the evaluation of conditions similar to real-world conditions with controlled variables. In this case, ANSYS software was used to analyze the mechanical behavior of the asphalt specimen and its interaction with the applied loads.

**Figure 3:**
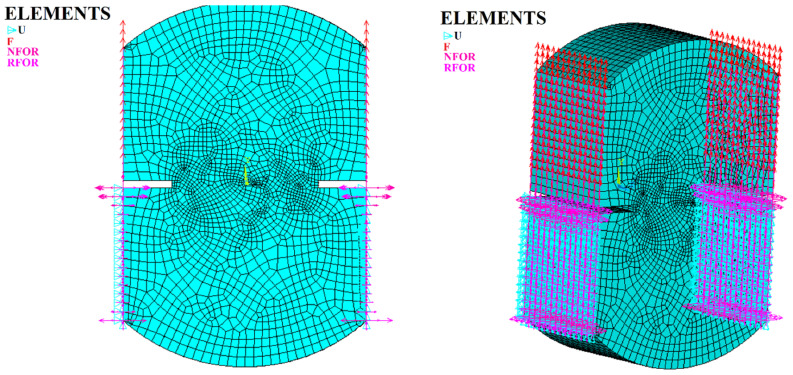
2D and 3D specimen geometries, displacement restrictions, and loading conditions.

To perform a simulation, the physical conditions of the model must be recreated. ANSYS software uses the finite element method (FEM), which enables the representation of the geometry of the specimens, and in particular uses three-dimensional modeling, which is a fundamental component of the solution. In this system, point, line, area, or solid elements were used, which can be intermixed. ANSYS also permits the generation of symmetric geometries and the establishment of boundary conditions, including the physical-mechanical properties of the elements, degrees of freedom, displacement constraints and the application of loads, to be applied in the simulation of the direct tension test (see Figure 3). The above enables obtaining and analyzing the results to directly relate the theoretical conditions to the real conditions presented in the test. Therefore, for this analysis, the SOLID185 finite element was chosen as the ideal element since it is a solid three-dimensional element with three degrees of freedom at each node and translations in the X, Y, and Z nodal directions. This element supports plasticity, hyperplasticity, tensile stiffness, creep, large deflection, and deformation capacities (see Figure 3).

Figure 3 shows the symmetric geometry creation in the ANSYS environment. It can be seen how the “U” elements that restrict the horizontal and vertical displacements of the FE nodes were placed at the bottom of the specimen, simulating the restraining effect of the experimental test clamping mechanism. Likewise, “U” elements restricting the horizontal displacement of the specimen were also placed at the top (but were omitted in Figure 3 for better visualization). Additionally, the loads (red arrows) were only applied to the top part of the specimen. All the above features were consistent with the characteristics of the experimental test.

**Figure 4:**
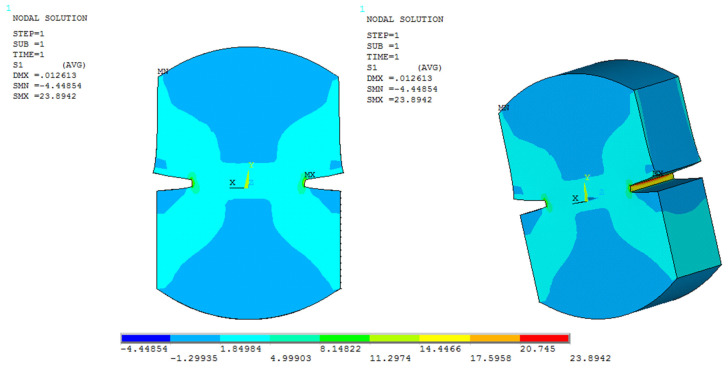
2D and 3D models of the stresses generated in the specimen.

Furthermore, to compare the numerical results obtained in ANSYS with those obtained in the experimental direct tension tests, a nodal analysis was performed on the 3D model of the specimen to simulate the stresses acting inside the specimen at the time when the maximum load is applied and before the fracture occurs (see Figure 4).

On the other hand, experimental development was used to determine the relevance, repeatability, and sensitivity of the test. This made it possible to identify the ideal conditions for the FEM model to function properly. As a result, specific tests were both carried out experimentally and simulated to establish the validation of the FEM model used to simulate the direct tension test.

In the classical theory of elasticity, the mechanical properties of elastic solids are considered in Hooke’s law; however, since asphalt mixtures are viscoelastic materials, Maxwell’s viscoelastic model was integrated to correctly validate the FEM model that simulates the direct tension test. Due to the ANSYS software environment, this model was inserted by means of a matrix, with the shear modulus (G) in kg/cm^2^ and the compressibility modulus (K) in kg/cm^2^ considered in columns C46–C50 in the program. These parameters were obtained from the experimental direct tension tests carried out in the laboratory.

## 3. Results and Discussion

**Figure 5:**
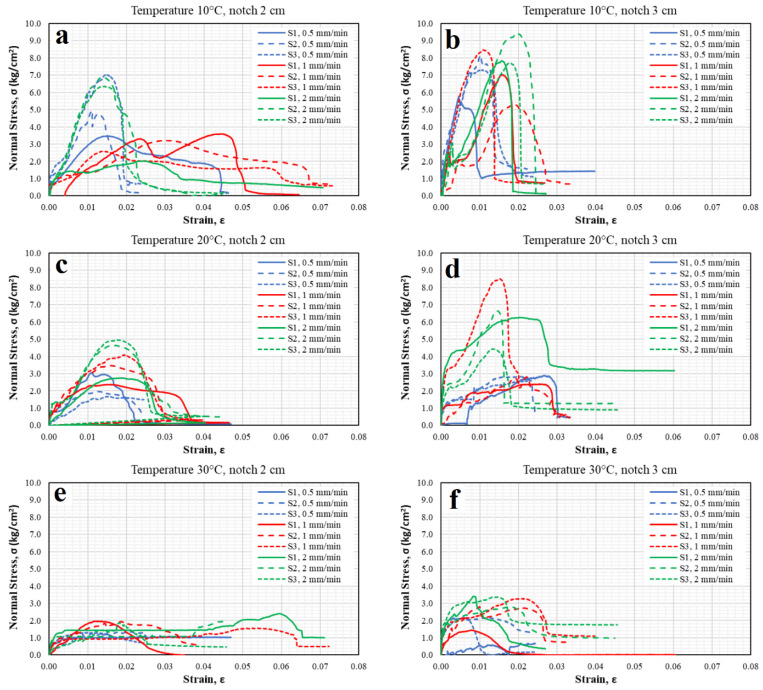
Normal stress-strain curves of specimens tested at different temperatures, load application speeds, and notch lengths.

**Figure 6:**
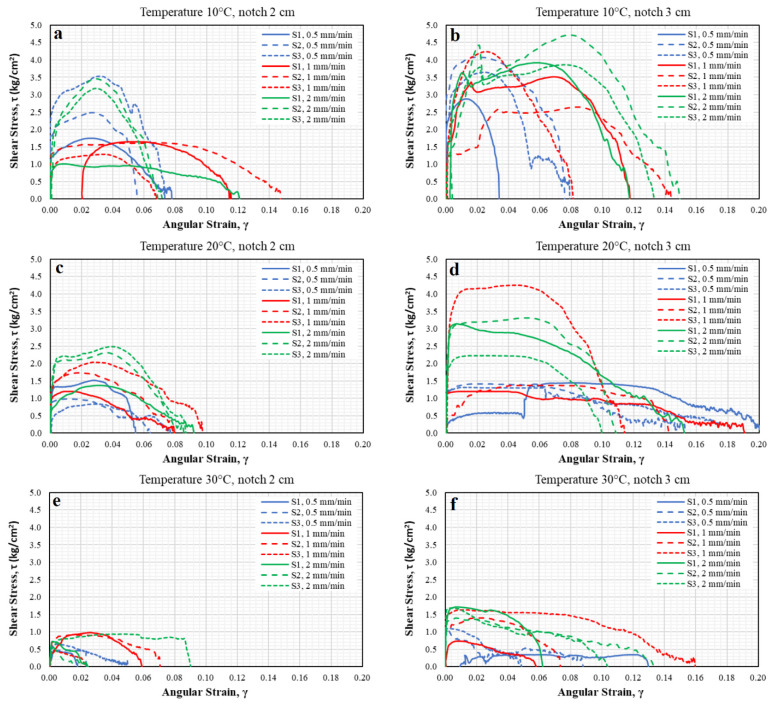
Shear stress-angular strain curves of specimens tested at different temperatures, load application speeds, and notch lengths.

Figure 5 shows the normal stress and unitary strain states, while Figure 6 shows the shear stress and angular strain states. According to Figure 5a, which corresponds to a specimen with a 2 cm notch tested at intermediate temperature of 10 °C and different load application rates, it is evident that the elastic behavior of the asphalt material dominates (greater stiffness in the samples); although a higher stress state is obtained, failure occurs in a fragile form, with no opportunity for recoverable strains. For specimens tested at a temperature of 30 °C (see Figure 5e), in most specimens, the viscous component of the material was significantly present. It means that, at very slow rates (0.5 mm/min), the viscous component dominates, diminishing the resistance to normal stress; in addition, permanent strains were present. However, at rates of 1 mm/min, the behavior of some specimens is close to the viscoelastic one. On the other hand, for the application of the fastest rate (2 mm/min), the specimens behave in a quasi-fragile manner. This variability is influenced by temperature, rate, and type of loading [60,61]. Therefore, this condition was discarded and not considered a good representation option. The above coincides as described by Saha and Biligiri [38] since the increase in temperature can reduce the elastic properties of the binder, which becomes viscous [19], while the reduction in temperature decreases the performance of the asphalt mix [42], which makes the binder brittle and the asphalt mix vulnerable to cracking [12,19]. For specimens tested at a temperature of 20 °C (see Figure 5c) at different load application rates, it was observed that the specimens adopted both components of the asphalt material (elastic and plastic), thus presenting a quasi-fragile behavior, which was ideal for evaluating cracking, as it was consistent with the objective of representing the real conditions to which a flexible pavement is subjected. The above described showed that 20 °C is the temperature at which the direct tension test performs best; this is consistent with Kim [60], as he specifies that asphalt undergoes thermal cracking at intermediate temperatures (5 to 45 °C), in which the asphalt exhibits viscoelastic behavior. 

In addition, for the optimal temperature choice, the notch size was determined based on the behavior during the tests. Figure 5b,d,f show that, during the tests (at different load rates), high dispersion is present since the specimens show low strain level under high stresses or vice versa, as well as staggering with sudden and gradual increases or decreases in resistance. This is probably because the material underwent structural rearrangements, should the thick parts be in contact with each other, which could provide sufficient internal friction due to the mechanical nature of the asphalt mixture. As a result, it can be inferred that the material did not develop uniformly, and under this selection parameter, the tests shown in Figure 5b,d,f exhibit a high variability level. Thus, a temperature of 20 °C, a notch length of 2 cm, and a load application rate of 1 mm/min were selected because they better represent the stress state of the test; in addition, they consider the elastic and viscous components of the material. Thus, according to Eghbali et al. [20], it has been confirmed in this study that a 2 cm notch in a 5 cm thick specimen is adequate, while a loading rate of 1 mm/min is ideal, which confirms that described by Fuan et al. [21], since the loading rate is between 1 and 6 mm/min. They concluded that lower temperatures and higher loading rates result in elastic behavior; in contrast, higher temperatures or lower loading rates result in viscous behavior of the binder.

After analyzing Figure 6, it is evident that the mechanical behavior was affected at this temperature, resulting in dispersion in the results. At a temperature of 10 °C, the specimen was stiffer and displayed high dispersion. Similarly, at a temperature of 30 °C, the dispersions were significant, demonstrating that the viscous behavior of the mixture dominated. On the other hand, observing the stress-strain curves of the specimens tested at 20 °C with 2 cm notches, dispersion is present, but at a lower magnitude. Thus, at this temperature, the asphalt mixes show typical viscoelastic behavior. Therefore, it better reflects the service conditions of asphalt pavements subjected to this temperature (intermediate). 

Once the ideal conditions for the direct tension test were found (20 °C temperature, 2 cm notch length and 1 mm/min load application rate), which reflect the working conditions of asphalt pavement, experimental tests and simulations were carried out in the ANSYS environment using the SOLID185 finite element. To start the process, the symmetric geometry of the specimen was created in 3D, and a volume mesh was established so that the software would recognize the specimen as a solid. Then, each node was assigned three degrees of freedom. In addition, the boundary conditions were established to simulate the direct tension test, with the vertical and horizontal displacements of the bottom part of the specimen (below the notches) restricted and the horizontal displacement of the top part of the specimen (above the notches) restricted, as shown in Figure 3. Additionally, the loading conditions (upward) that produce cracking in the specimen were placed at the top of the specimen. Finally, the load application speed was assigned.

**Figure 7:**
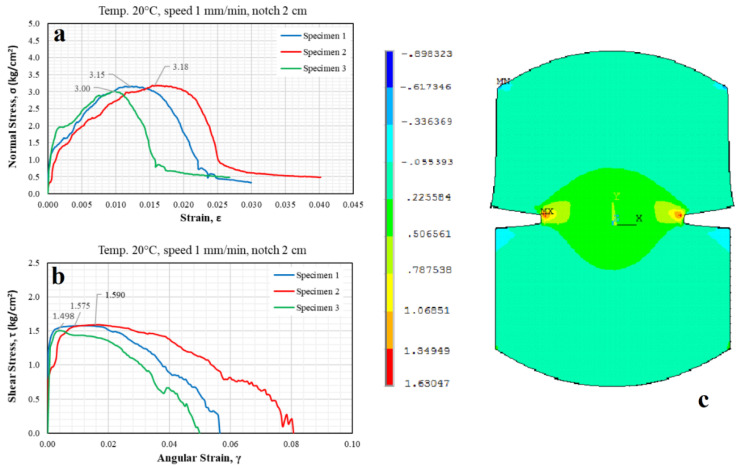
Normal and shear stresses obtained from (**a**,**b**) experimental direct tension tests and (**c**) the simulations.

Figure 7a shows that the maximum normal stresses supported by specimens 1, 2, and 3 in the direct tension test were 3.15, 3.18, and 3.00 kg/cm^2^, respectively. These stresses act perpendicular to the XZ plane, corresponding to the plane of the surface with a notch formed by the two 2 cm notches produced in the specimen. On the other hand, Figure 7b shows that the maximum shear stresses that produced cracking in the failure plane were 1.575, 1.590, and 1.498 kg/cm^2^. Additionally, based on the simulations, the shear strengths of the specimens tested under ideal conditions were 1.63, 6.48, and 1.57 kg/cm^2^ for the XZ, XY, and YZ planes, respectively.

Therefore, comparing the coordinate system used in the model with the experimental results, the maximum acting shear stress at the moment of failure in the XZ plane was the only stress that can be obtained from the direct tension test. When the analysis was performed in a plane stress state, the stresses were 1.575 kg/cm^2^, 1.590 kg/cm^2^, and 1.498 kg/cm^2^ for specimens 1, 2, and 3, respectively. These stresses are very similar to the stress of 1.63 kg/cm^2^ shown by the simulation under the same conditions, representing a variation of 8.12% between the experimental and simulated results.

It should be noted that the shear stress results obtained from the tests and simulations follow Mohr’s interpretation, which states the maximum shear stress in the plane during the uniaxial tension test is equal to 50% of the normal stress value at the yield limit (Figure 7c). It means, from Figure 7, if a direct tension test is performed, a failure stress will be equal to the highest principal stress, which for the specimen would be the yield stress. However, since it is unconfined, the intermediate and minor principal stresses are zero. Therefore, there is only one Mohr’s circle that is tangent to the origin. Then, at failure, if the major principal stress is equal to the yield stress or rupture stress (normal stress specimen 1 = 3.15 kg/cm^2^), the value of the maximum shear stress that caused the failure is the radius of the circle and equal to half of the major principal stress (shear stress specimen 1 = 1.575 kg/cm^2^). However, according to the model, the fracture caused by the maximum shear stress appeared much earlier in a plane other than the plane considered in the analysis.

Additionally, Pearson’s Coefficient of Variation (CV) was calculated from the results shown in Figure 7a,b. The CV obtained from the ratio of normal stress to strain was 3.10%, while, for the relationship of shear stress to angular strain, it was 3.18%. This indicates that the dispersion of the results is minimal, implying that the indirect tension test presents an adequate repeatability. 

**Figure 8:**
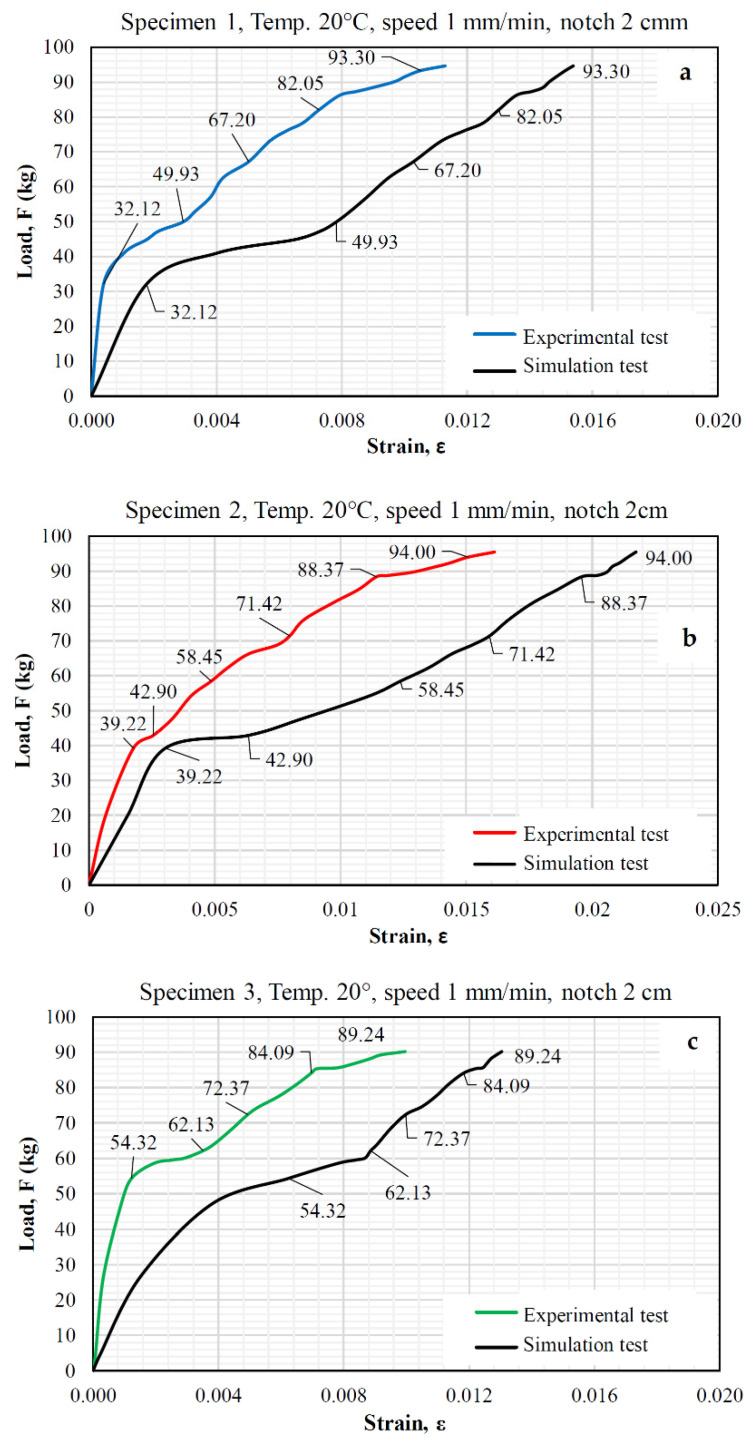
Load-strain curves obtained from the simulations and experimental direct tension tests.

Furthermore, Figure 8 shows a comparison between the load-strain curves of the simulations and the experimental direct tension tests. Based on Figure 8, it is evident that the results of the cracking behavior of the asphalt specimens in the direct tension test and the simulation of the proposed model varied by 30% to 35%. This result was obtained using the indirect result error calculation method, which analyzes two databases with different magnitudes that are related to one another. Therefore, it was established that the direct tension test results and the simulations were relatively consistent (see Figure 8); thus, the FEM model is congruent, since the simulation environment represents the operational conditions of an asphalt pavement because the elements are considered continuous media.

From Figure 8, the FEM model predicts higher than expected strains because it considers a continuous homogeneous medium with constant viscoelastic properties throughout the medium and a linear viscoelastic behavior, under ideal conditions, and the real specimen is a material composed of stone and asphalt materials, where the individual properties of each of them are clearly different; in addition, the mechanical interlocking of the angular materials causes the model to behave with greater stiffness, which can be observed in Figure 8, as the behavior curves of the simulation are shifted to the right. Hence, in a more advanced mechanical model, which considers the composite with the individual properties of the materials and the conditions of stress transmission through the contact points, where the normal and shear stresses are concentrated, the results could be more approximate, and the prediction error could be reduced.

**Figure 9:**
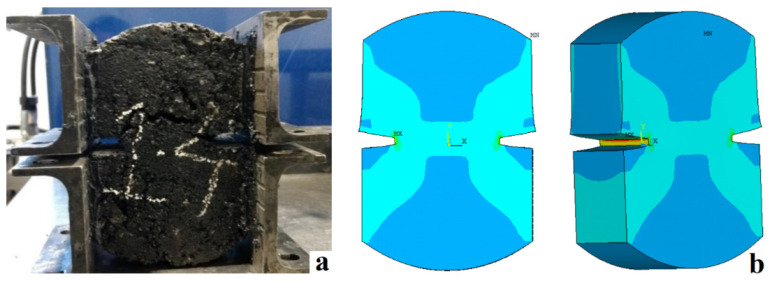
Failure planes: (**a**) on the experimental asphalt specimen, (**b**) on the 2D and 3D simulated specimens.

Additionally, based on the evaluation and application of the mechanical properties obtained from the different experimental tests on the specimens and the simulations, the results show a correct relationship between the physical tests (experimental) and those analyzed with software (simulations) since the failure planes and behavior are clearly similar (see Figure 9), indicating that the model is suitable for estimating and predicting the cracking behavior of asphalt pavement.

## 4. Conclusions

In this paper, the mode I fracture toughness of asphalt mixtures was studied, considering symmetrical circular specimens cut and notched at their edges to induce a fracture in the central notch. These were subjected to direct tension tests at intermediate temperatures of 10, 20, and 30 °C, mode I load rates (0.5, 1, and 2 mm/min) and notches (2 and 3 cm) to find the variables that reflect the operating conditions of the asphalt mix and finally validate a FEM model. Based on this research, conclusions are derived as follows:

The specimens with 2 cm notches tested at a temperature of 10 °C with various load application speeds showed the elastic behavior of the asphalt material, exhibiting greater stiffness due to the higher stress state, producing a fragile failure. On the other hand, specimens tested at a temperature of 30 °C showed the viscous component of the material since the normal stress was lower, producing permanent strains. Therefore, these conditions are not representative and were discarded. In contrast, the specimens tested at a temperature of 20 °C at different load application speeds showed both components of the asphalt material (elastic and plastic) and demonstrated a quasi-fragile behavior, which is representative of the real conditions to which a pavement is subjected and is thus ideal for evaluating cracking.

In terms of the length of the notches and the load application speed, it can be seen that the material experienced structural rearrangements since the coarse particles were in contact with each other, providing sufficient internal friction due to the mechanical nature of the asphalt mixture. Therefore, it can be inferred that the material did not develop uniformly. Thus, a temperature of 20 °C, a notch length of 2 cm, and a load application speed of 1 mm/min were the best conditions for representing the stress state of the test; in addition, these conditions consider both the elastic and viscous components of the material.

When considering the plane stress state in the direct tension test, the shear stress in the XZ plane was known; thus, the shear stress results obtained from the simulations were found to be less than 8.12% different than the experimental results. This establishes that the FEM model adequately represents the direct tension test.

When the load-strain curves of the simulations and the experimental direct tension tests were analyzed, it was evident that the variation between the cracking behavior of the asphalt specimens in the direct tension test and the simulation of the proposed model varied by 30% to 35%. Additionally, the failure planes and behavior were similar; therefore, it can be concluded that the FEM model is adequate for estimating and predicting the cracking behavior of asphalt mixture, since the FEM model is congruent, and the simulation environment represents the operational conditions of an asphalt mixture when the elements were considered to be continuous media.

Finally, the limitations of the model prevent it from producing results as accurate as those obtained in experimental tests because the specimen is treated as a continuous medium without accounting for discontinuities between the aggregates and asphalt, and the rearrangement of the granular medium during the testing process greatly contributes to the variability of the results.

## 5. Future Work

Perform tests with traditional methods under conditions similar to those used in this study for a comparative analysis between this indirect tension test and traditional methods;

Study specimens with void variations, different types of asphalt material, and variations in the size distribution of their coarse aggregate;

Include an analysis with new types of pavements, and agents such as RAP, polymers, and others added, with the aim of evaluating the model and the direct tension test using variations of the materials under the same conditions used in this study (temperature, load application speed, and notch length);

Perform additional direct tension tests to obtain results that better reflect the mechanical properties and incorporate these results into the model to establish a more accurate correlation to verify the viability and feasibility of the test.

## Figures and Tables

**Table 1:** Characteristics of the coarse aggregate.

Characteristic	Normative	Value Obtained	Specification PA-MA-001/2008
Abrasion and Impact in the Los Angeles Machine, %	ASTM C131-03	10%	30% max. (structural layers)
Degradation by Abrasion in the Micro-Deval Apparatus, %	ASTM D 6928-03	7%	18% max. (structural layers)
Soundness of Aggregates by Use of Sodium Sulfate, %	ASTM C88-90	1.12%	15% max.
Percentage of Fractured Particles in Coarse Aggregate, % (2 faces or more)	ASTM D 5821	98%	90% min.
Elongated Particles in Coarse Aggregate, %	ASTM D 4791	1.60%	5 a 1%, 10% max.
Flat Particles in Coarse Aggregate, %	ASTM D 4791	0.34%	5 a 1%, 10% max.
Adherence with the asphalt, % of covering	Recommendation AMAAC RA-08/2008	96%	90% min.

**Table 2:** Characteristics of the fine aggregates.

Test	Normative	Value Obtained	Specification PA-MA-001/2008
Equivalent Value of Fine Aggregate, %	ASTM D 2419	70%	50% min. (structural layers)
Angularity, %	AASHTO T 304	41%	40% min.
Methylene blue, g/g	Recommendation AMAAC RA-05/2010	14	15% max. (structural layers)

**Table 3:** Experimental design.

Variables						
Temperature (°C)	10		20		30	
Notch length (cm)	2	3	2	3	2	3
Load application rate (mm/min)	0.5	0.5	0.5	0.5	0.5	0.5
	1	1	1	1	1	1
	2	2	2	2	2	2

## Data Availability

All data included in this study are available upon request by contact with the corresponding author.
